# New classes of difference cum-ratio-type exponential estimators for a finite population variance in stratified random sampling

**DOI:** 10.1016/j.heliyon.2024.e33402

**Published:** 2024-06-27

**Authors:** Umer Daraz, Jinbiao Wu, Mohammed Ahmed Alomair, Luai Abdulla Aldoghan

**Affiliations:** a School of Mathematics and Statistics, Central South University, Changsha 410017, China; b Department of Quantitative Methods, School of Business, King Faisal University, Al-Ahsa 31982, Saudi Arabia

**Keywords:** Exponential estimators, Variance estimation, Minimum and maximum values, Stratified random sampling, *PRE*

## Abstract

The problem of estimating the variance of a finite population is an important issue in practical situations where controlling variability is difficult. During experiments conducted in the fields of agriculture and biology, researchers often face this issue, resulting in outcomes that appear uncontrollable for the desired results. Using auxiliary information effectively has the potential to enhance the precision of estimators. This article aims to introduce improved classes of efficient estimators that are specifically designed to estimate the study variable's finite population variance. When stratified random sampling is used, these estimators are particularly efficient when the minimum and maximum values of the auxiliary variable are known. The bias and mean squared error (*MSE*) of the proposed classes of estimators are determined by a first-order approximation. In order to evaluate their performance and verify the theoretical results, we performed simulation research. The proposed estimators show higher percent relative efficiencies (PREs) in all simulation scenarios compared to other existing estimators, according to the results. Three datasets are utilized in the application section, which are used to further validate the effectiveness of the proposed estimators.

## 1 Introduction

In order to optimize the performance of the estimators under study while minimizing costs, time, and effort, survey sampling aims to get complete details on particular characteristics of the population. In many populations, there are a few extreme observations, and estimating unknown population parameters without considering this data can be highly sensitive. In such cases, the outcomes may either be underestimated or overestimated. However, it is important to note that the efficiency of estimators usually declines in terms of mean square error when confronted with extreme values in the data set. Removing such data from the sample may be tempting. To successfully address this difficulty, it is extremely important to include this information in the process of determining population characteristics. By transforming the known largest and smallest observations of the auxiliary variable using a linear transformation, [20] introduced two estimators. After that, these concepts were not investigated any further until the ideas of [17]. They used the technique of applying the largest and smallest observations of the auxiliary variable to different finite population mean estimators. [18] optimized the estimate of the finite population mean under largest and smallest observations using a two-phase sampling method. For more details, see [2], [3], [4], [8], [9], [11], [12], [19], [26] and references therein.

The problem of estimating the variance of a finite population is an important issue in practical situations where controlling variability is difficult. During experiments conducted in the fields of agriculture and biology, researchers often face this issue, resulting in outcomes that appear uncontrollable for the desired results. Using auxiliary information effectively has the potential to enhance the precision of estimators. In this paper, we propose two enhanced classes of estimators for finite population variance estimation that use the known extreme values of the auxiliary variable for further improvement, as discussed by [11], [12], [13]. To some extent, stratified sampling can help us deal with variability in the planning, design, and estimation phases. Using stratified sampling, homogeneous groups known as strata are generated from different groups, and samples can be taken from each stratum independently. A lot of researchers have worked on different types of estimators to estimate the finite population variance, including [1], [5], [6], [14], [15], [16], [21], [22], [23], [24], [27], [28], [29].

Extreme values of the auxiliary variable are used as auxiliary information in this article, and are retained in the data. With a stratified random sampling technique, we propose two enhanced classes of estimators for finite population variance estimation that use the known extreme values of the auxiliary variable.

This article is divided into the following sections. The notations and methods are introduced in Section 2. Section 3 covers some existing estimators. We provide comprehensive detail on our suggested estimators in Section 4. The mathematical comparison is given in Section 5. In Section 6, a simulation study is presented to produce six distinct artificial populations using various probability distributions in order to validate the theoretical outcomes covered in Section 5. This section also includes some numerical examples to further clarify our theoretical findings. Finally, some conclusions and recommendations for further research are included in Section 7.

## 2 Notations and symbols

Consider a finite population $\delta =\left(\delta_{1},\delta_{2},\ldots ,\delta_{N}\right)$ of size *N*, that is divided into *L* strata, each of size $N_{h}(h=1,2,\ldots ,L)$, such that $\sum_{h=1}^{L}N_{h}=N$. Let the values of the study variable (*Y*) and the auxiliary variable (*X*) in the $h^{th}$ stratum of the $i^{th}(i=1,2,\ldots ,N_{L})$ unit be $y_{hi}$ and $x_{hi}$, respectively.

A sample of size $n_{h}$ is selected from the $h^{th}$ stratum through simple random sampling without replacement. Let $\overset{¯}{Y_{h}}=\left(1/N_{h}\right)\sum_{h=1}^{L}Y_{hi}$ and $\overset{¯}{X_{h}}=\left(1/N_{L}\right)\sum_{h=1}^{L}X_{hi}$ represent the population means of the research variable as well as the supplementary variable in the $h^{th}$ stratum that corresponding to the population means

$\overset{¯}{Y}=\frac{1}{N_{h}}\sum_{h=1}^{L}W_{h}\overset{¯}{Y_{h}}$ and $\overset{¯}{X}=\frac{1}{N_{h}}\sum_{h=1}^{L}W_{h}\overset{¯}{X_{h}}$

respectively, where the known stratum weight is denoted by $W_{h}=N_{h}/N$. Let

$S_{yh}^{2}=\frac{\sum_{i=1}^{N_{h}}{\left(Y_{hi}-\overset{¯}{Y_{h}}\right)}^{2}}{N_{h}-1}$ and $S_{xh}^{2}=\frac{\sum_{i=1}^{N_{h}}{\left(X_{hi}-\overset{¯}{X_{h}}\right)}^{2}}{N_{h}-1}$

be the population variances in the $h^{th}$ stratum. Let $\overset{¯}{y_{h}}$ and $\overset{¯}{x_{h}}$ be the sample means of the study and the auxiliary variables in the $h^{th}$ stratum, and their corresponding sample variances are

$s_{yh}^{2}=\frac{\sum_{i=1}^{n_{h}}{\left(y_{hi}-\overset{¯}{Y_{h}}\right)}^{2}}{n_{h}-1}$ and $s_{xh}^{2}=\frac{\sum_{i=1}^{n_{h}}{\left(x_{hi}-\overset{¯}{x_{h}}\right)}^{2}}{n_{h}-1}$,

respectively.

The following terms are defined in order to calculate the biases and mean square errors for different estimators:

$e_{0h}=\left(\frac{s_{yh}^{2}-S_{yh}^{2}}{S_{yh}^{2}}\right)$, $e_{1h}=\left(\frac{s_{xh}^{2}-S_{xh}^{2}}{S_{xh}^{2}}\right)$, $e_{2h}=\left(\frac{\overset{¯}{x_{h}}-\overset{¯}{X_{h}}}{\overset{¯}{X_{h}}}\right)$,

such that $E\left(e_{ih}\right)=0$ for i=0,1,2.

$$E\left(e_{0h}^{2}\right)=\theta_{h}\lambda_{40h}^{⁎},\;E\left(e_{1h}^{2}\right)=\theta_{h}\lambda_{04h}^{⁎},\;E\left(e_{2h}^{2}\right)=\theta_{h}C_{xh}^{2},$$

$$E\left(e_{0h}e_{1h}\right)=\theta_{h}\lambda_{22h}^{⁎},\;E\left(e_{0h}e_{2h}\right)=\theta C_{xh}\lambda_{21h},\;E\left(e_{1h}e_{2h}\right)=\theta_{h}C_{xh}\lambda_{03h},$$

where $\lambda_{40h}^{⁎}=(\lambda_{40h}-1)$, $\lambda_{04h}^{⁎}=(\lambda_{04h}-1)$, $\lambda_{22h}^{⁎}=(\lambda_{22h}-1)$, $\theta_{h}=\left(\frac{1}{n_{h}}-\frac{1}{N_{h}}\right)$.

Also

$$\lambda_{rsh}=\frac{\mu_{rsh}}{\mu_{20h}^{r/2}\mu_{02h}^{s/2}},$$

where

$$\mu_{rsh}=\frac{\sum_{i=1}^{N_{h}}{\left(Y_{hi}-\overset{¯}{Y_{h}}\right)}^{r}{\left(X_{hi}-\overset{¯}{X_{h}}\right)}^{s}}{N_{h}-1}.$$

Here, $\lambda_{40h}=\beta_{2(yh)}$ and $\lambda_{04h}=\beta_{2(xh)}$.

## 3 Existing estimators

This section introduces the existing estimators of finite population variances.

In stratified random sampling, the variance of the usual estimator $\overset{¯}{y_{st}}=\sum_{h=1}^{L}W_{h}\overset{¯}{y_{h}}$, is defined as follows

$$Var(\overset{¯}{y_{st}})=\sum_{h=1}^{L}\theta_{h}W_{h}^{2}S_{yh}^{2}=S_{yst}^{2}.$$

The unbiased estimator ${\overset{ˆ}{S}}_{yst}^{2}$ of $S_{yst}^{2}$, is defined as

$${\overset{ˆ}{S}}_{yst}^{2}=\sum_{h=1}^{L}\theta_{h}W_{h}^{2}s_{yh}^{2}.$$

The usual variance estimator of ${\overset{ˆ}{S}}_{yst}^{2}=s_{yst}^{2}$ for population variance is given by

(3.1) $$Var({\overset{ˆ}{S}}_{yst}^{2})=\sum_{h=1}^{L}\theta_{h}^{3}W_{h}^{4}S_{yh}^{4}\lambda_{40h}^{⁎}.$$

[15] suggested a ratio estimator for population variance ${\overset{ˆ}{S}}_{Rst}^{2}$, which is given by

(3.2) $${\overset{ˆ}{S}}_{Rst}^{2}=\sum_{h=1}^{L}\theta_{h}W_{h}^{2}s_{yh}^{2}\left(\frac{S_{xh}^{2}}{s_{xh}^{2}}\right).$$

The following expressions represent the bias and *MSE* of ${\overset{ˆ}{S}}_{Rst}^{2}$

(3.3) $$Bias\left({\overset{ˆ}{S}}_{Rst}^{2}\right)≅\sum_{h=1}^{L}\theta_{h}^{2}W_{h}^{2}S_{yh}^{2}\left(\lambda_{04h}^{⁎}-\lambda_{22h}^{⁎}\right)$$

and

(3.4) $$MSE\left({\overset{ˆ}{S}}_{Rst}^{2}\right)≅\sum_{h=1}^{L}\theta_{h}^{3}W_{h}^{4}S_{yh}^{4}\left(\lambda_{40h}^{⁎}+\lambda_{04h}^{⁎}-2\lambda_{22h}^{⁎}\right).$$

The separate linear regression estimator ${\overset{ˆ}{S}}_{lrs}^{2}$, is defined as

(3.5) $${\overset{ˆ}{S}}_{lrst}^{2}=\sum_{h=1}^{L}\theta_{h}W_{h}^{2}\left[s_{yh}^{2}+b_{\left(s_{yh}^{2},s_{xh}^{2}\right)}\left(S_{xh}^{2}-s_{xh}^{2}\right)\right],$$

where $b_{\left(s_{yh}^{2},s_{xh}^{2}\right)}=\frac{s_{yh}^{2}\lambda_{22h}^{⁎}}{s_{xh}^{2}\lambda_{04h}^{⁎}}$, is the sample regression coefficient.

The following expression represents the *MSE* of ${\overset{ˆ}{S}}_{lrst}^{2}$

(3.6) $$MSE\left({\overset{ˆ}{S}}_{lrst}^{2}\right)≅\sum_{h=1}^{L}\theta_{h}^{3}W_{h}^{4}S_{yh}^{4}\lambda_{40h}^{⁎}\left(1-\rho_{h}^{⁎2}\right),$$

where $\rho_{h}^{⁎}=\frac{\lambda_{22h}^{⁎}}{\sqrt{\lambda_{40h}^{⁎}}\sqrt{\lambda_{04h}^{⁎}}}$.

[6] introduced an exponential ratio type estimator ${\overset{ˆ}{S}}_{BTst}^{2}$, is defined as

(3.7) $${\overset{ˆ}{S}}_{BTst}^{2}=\sum_{h=1}^{L}\theta_{h}W_{h}^{2}\left[s_{yh}^{2}\exp\left(\frac{S_{xh}^{2}-s_{xh}^{2}}{S_{xh}^{2}+s_{xh}^{2}}\right)\right].$$

The following expressions represent the bias and *MSE* of ${\overset{ˆ}{S}}_{BTst}^{2}$

(3.8) $$Bias\left({\overset{ˆ}{S}}_{BTst}^{2}\right)≅\frac{1}{2}\sum_{h=1}^{L}\theta_{h}^{2}W_{h}^{2}S_{yh}^{2}\left(\frac{3\lambda_{04h}^{⁎}}{4}-\lambda_{22h}^{⁎}\right)$$

and

(3.9) $$MSE\left({\overset{ˆ}{S}}_{BTst}^{2}\right)≅\sum_{h=1}^{L}\theta_{h}^{3}W_{h}^{4}S_{yh}^{4}\left(\lambda_{40h}^{⁎}+\frac{\lambda_{04h}^{⁎}}{4}-\lambda_{22h}^{⁎}\right).$$

[25] proposed a ratio-type estimator ${\overset{ˆ}{S}}_{USst}^{2}$, which is express as

(3.10) $${\overset{ˆ}{S}}_{USst}^{2}=\sum_{h=1}^{L}\theta_{h}W_{h}^{2}s_{yh}^{2}\left(\frac{S_{xh}^{2}+\lambda_{04h}}{s_{xh}^{2}+\lambda_{04h}}\right).$$

The following expressions represent the bias and *MSE* of ${\overset{ˆ}{S}}_{USst}^{2}$

(3.11) $$Bias\left({\overset{ˆ}{S}}_{USst}^{2}\right)≅\sum_{h=1}^{L}\theta_{h}^{2}W_{h}^{2}S_{yh}^{2}g_{0h}\left(g_{0h}\lambda_{04h}^{⁎}-\lambda_{22h}^{⁎}\right)$$

and

(3.12) $$MSE\left({\overset{ˆ}{S}}_{USst}^{2}\right)≅\sum_{h=1}^{L}\theta_{h}^{3}W_{h}^{4}S_{yh}^{4}\left(\lambda_{40h}^{⁎}+g_{0h}^{2}\lambda_{04h}^{⁎}-2g_{0h}\lambda_{22h}^{⁎}\right),$$

where $g_{0h}=\frac{S_{xh}^{2}}{S_{xh}^{2}+\lambda_{04h}}$.

[16] suggested some ratio estimators ${\overset{ˆ}{S}}_{KCist}^{2}$ are defined as

(3.13) $${\overset{ˆ}{S}}_{KC1st}^{2}=\sum_{h=1}^{L}\theta_{h}W_{h}^{2}s_{yh}^{2}\left(\frac{S_{xh}^{2}+C_{xh}}{s_{xh}^{2}+C_{xh}}\right),$$

(3.14) $${\overset{ˆ}{S}}_{KC2st}^{2}=\sum_{h=1}^{L}\theta_{h}W_{h}^{2}s_{yh}^{2}\left(\frac{\lambda_{04h}S_{xx}^{2}+C_{xh}}{\lambda_{04h}s_{xh}^{2}+C_{xh}}\right),$$

(3.15) $${\overset{ˆ}{S}}_{KC3st}^{2}=\sum_{h=1}^{L}\theta_{h}W_{h}^{2}\left(\frac{C_{xh}S_{xh}^{2}+\lambda_{04h}}{C_{xh}s_{xh}^{2}+\lambda_{04h}}\right),$$

where $C_{xh}=\frac{S_{xh}}{\overset{¯}{X_{h}}}$ is the population coefficient of variation.

The bias and $MSE^{\prime }s$ of ${\overset{ˆ}{S}}_{KCist}^{2}(i=1,2,3)$ are expressed as follows

(3.16) $$Bias\left({\overset{ˆ}{S}}_{KCist}^{2}\right)≅\sum_{h=1}^{L}\theta_{h}^{2}W_{h}^{2}S_{yh}^{2}g_{ih}\left(g_{ih}\lambda_{04h}^{⁎}-\lambda_{22h}^{⁎}\right)$$

and

(3.17) $$MSE\left({\overset{ˆ}{S}}_{KCist}^{2}\right)≅\sum_{h=1}^{L}\theta_{h}^{3}W_{h}^{4}S_{yh}^{4}\left(\lambda_{40h}^{⁎}+g_{ih}^{2}\lambda_{04h}^{⁎}-2g_{ih}\lambda_{22h}^{⁎}\right),$$

where $g_{1h}=\frac{S_{xh}^{2}}{S_{xh}^{2}+C_{xh}},\;g_{2h}=\frac{\lambda_{04h}S_{xh}^{2}}{\lambda_{04h}S_{xh}^{2}+C_{xh}},\;g_{3h}=\frac{C_{xh}S_{xh}^{2}}{C_{xh}S_{xh}^{2}+\lambda_{04h}}$.

## 4 Proposed estimators

This section introduces some improved classes of estimators inspired by [11], [12], [13] that use the known largest and smallest values of the auxiliary variables to calculate the finite population variance. The suggested estimators are defined as

(4.1) $${\overset{ˆ}{S}}_{Dst}^{2}=\sum_{h=1}^{L}\theta_{h}W_{h}^{2}\left[k_{1h}s_{yh}^{2}{\left(\frac{S_{xh}^{2}}{s_{xh}^{2}}\right)}^{\alpha_{1}}+k_{2h}\left(\overset{¯}{X_{h}}-\overset{¯}{x_{h}}\right){\left(\frac{S_{xh}^{2}}{s_{xh}^{2}}\right)}^{\alpha_{2}}\right]\exp\left[\frac{a_{1h}(S_{xh}^{2}-s_{xh}^{2})}{a_{1h}(s_{xh}^{2}+S_{xh}^{2})+2a_{2h}}\right],$$

(4.2) $${\overset{ˆ}{S}}_{Dest}^{2}=\sum_{h=1}^{L}\theta_{h}W_{h}^{2}s_{yh}^{2}\exp\left[k_{3h}\left\{\frac{\left(\overset{¯}{X_{h}}-\overset{¯}{x_{h}}\right)}{\left(\overset{¯}{X_{h}}+\overset{¯}{x_{h}}\right)+2b_{1h}}\right\}\right]\exp\left[k_{4h}\left\{\frac{\left(S_{xh}^{2}-s_{xh}^{2}\right)}{\left(S_{xh}^{2}+s_{xh}^{2}\right)+2b_{1h}}\right\}\right]$$

where the scalar quantities ($\alpha_{1},\alpha_{2}$) contain the values (0,−1,1), $\left(k_{ih},i=1,2\right)$ are unknown constants whose values need to be determined so as to minimize the biases and the mean squared errors, and $\left(k_{3h}=k_{4h}=1\right)$. The parameters of the auxiliary variable are $a_{1h}$, $a_{2h}$, and $b_{1h}=X_{Mh}-X_{mh}$. The different classes of the proposed estimator (I) that we derive from (4.1) are presented in 1

**Table 1:** Different classes of the suggested estimator-I.

Members of the suggested estimator ${\overset{ˆ}{S}}_{Dst}^{2}$	*α*_1_	*α*_2_	*a*_1*h*_	*a*_2*h*_
${\overset{ˆ}{S}}_{D1}^{2}=\sum_{h=1}^{L}\theta_{h}W_{h}^{2}\left[k_{1h}s_{yh}^{2}\left(\frac{S_{xh}^{2}}{s_{xh}^{2}}\right)+k_{2h}\left(\overset{¯}{X_{h}}-\overset{¯}{x_{h}}\right)\left(\frac{s_{xh}^{2}}{S_{xh}^{2}}\right)\right]L_{h}$	1	−1	−*β*_2(*xh*)_	*x*_*Mh*_ − *x*_*mh*_
${\overset{ˆ}{S}}_{D2}^{2}=\sum_{h=1}^{L}\theta_{h}W_{h}^{2}\left[k_{1h}s_{yh}^{2}\left(\frac{S_{xh}^{2}}{s_{xh}^{2}}\right)+k_{2h}\left(\overset{¯}{X_{h}}-\overset{¯}{x_{h}}\right)\right]L_{h}$	1	0	*x*_*Mh*_ − *x*_*mh*_	*c*_*xh*_
${\overset{ˆ}{S}}_{D3}^{2}=\sum_{h=1}^{L}\theta_{h}W_{h}^{2}\left[k_{1h}s_{yh}^{2}\left(\frac{s_{xh}^{2}}{S_{xh}^{2}}\right)+k_{2h}\left(\overset{¯}{X_{h}}-\overset{¯}{x_{h}}\right)\left(\frac{s_{xh}^{2}}{S_{xh}^{2}}\right)\right]L_{h}$	−1	−1	*x*_*Mh*_ − *x*_*mh*_	−*c*_*xh*_
${\overset{ˆ}{S}}_{D4}^{2}=\sum_{h=1}^{L}\theta_{h}W_{h}^{2}\left[k_{1h}s_{yh}^{2}\left(\overset{¯}{X_{h}}-\overset{¯}{x_{h}}\right)\left(\frac{S_{xh}^{2}}{s_{xh}^{2}}\right)\right]L_{h}$	0	1	*x*_*Mh*_ − *x*_*mh*_	*β*_2(*xh*)_
${\overset{ˆ}{S}}_{D5}^{2}=\sum_{h=1}^{L}\theta_{h}W_{h}^{2}\left[k_{1h}s_{yh}^{2}+k_{2}\left(\overset{¯}{X_{h}}-\overset{¯}{x_{h}}\right)\left(\frac{s_{xh}^{2}}{S_{xh}^{2}}\right)\right]L_{h}$	0	−1	*β*_2(*xh*)_	*x*_*Mh*_ − *x*_*mh*_
${\overset{ˆ}{S}}_{D6}^{2}=\sum_{h=1}^{L}\theta_{h}W_{h}^{2}\left[k_{1h}s_{yh}^{2}\left(\frac{S_{xh}^{2}}{s_{xh}^{2}}\right)+k_{2h}\left(\overset{¯}{X_{h}}-\overset{¯}{x_{h}}\right)\left(\frac{S_{xh}^{2}}{s_{xh}^{2}}\right)\right]L_{h}$	1	1	*x*_*Mh*_ − *x*_*mh*_	−*β*_2(*x*)_
${\overset{ˆ}{S}}_{D7}^{2}=\sum_{h=1}^{L}\theta_{h}W_{h}^{2}\left[k_{1h}s_{yh}^{2}\left(\frac{s_{xh}^{2}}{S_{xh}^{2}}\right)+k_{2h}\left(\overset{¯}{X_{h}}-\overset{¯}{x_{h}}\right)\left(\frac{S_{xh}^{2}}{s_{xh}^{2}}\right)\right]L_{h}$	−1	1	−*c*_*xh*_	*x*_*Mh*_ − *x*_*mh*_
${\overset{ˆ}{S}}_{D8}^{2}=\sum_{h=1}^{L}\theta_{h}W_{h}^{2}\left[k_{1h}s_{yh}^{2}\left(\frac{s_{xh}^{2}}{S_{xh}^{2}}\right)+k_{2h}\left(\overset{¯}{X_{h}}-\overset{¯}{x_{h}}\right)\right]\;L_{h}$	−1	0	*c*_*xh*_	*x*_*Mh*_ − *x*_*mh*_

where $L_{h}=\exp\left[\frac{a_{1h}(S_{xh}^{2}-s_{xh}^{2})}{a_{1h}(s_{xh}^{2}+S_{xh}^{2})+2a_{2h}}\right]$.

### 4.1 Properties of the suggested estimator-I

To obtain the bias and *MSE* of the first suggested estimator ${\overset{ˆ}{S}}_{Dst}^{2}$, we now rewrite (4.1) in terms of errors, i.e.

(4.3) $${\overset{ˆ}{S}}_{Dst}^{2}=\sum_{h=1}^{L}\theta_{h}W_{h}^{2}\left[k_{1h}S_{yh}^{2}\left(1+e_{0h}\right){\left(1+e_{1h}\right)}^{-\alpha_{1}}-k_{2h}\overset{¯}{X_{h}}e_{2h}{\left(1+e_{1h}\right)}^{-\alpha_{2}}\right]\exp\left[\frac{-g_{4h}e_{1h}}{2}{\left(1+\frac{g_{4h}}{2}e_{1h}\right)}^{-1}\right],$$

where $g_{4h}=\frac{a_{1h}S_{xh}^{2}}{a_{1h}S_{xh}^{2}+a_{2h}}$.

The following equation is obtained by employing the first-order Taylor series approximation

(4.4) $$\begin{array}{c} {\overset{ˆ}{S}}_{Dst}^{2}-\sum_{h=1}^{L}\theta_{h}W_{h}^{2}S_{yh}^{2}≅\sum_{h=1}^{L}\theta_{h}W_{h}^{2}[-S_{yh}^{2}+k_{1h}S_{yh}^{2}\{1+e_{0h}+e_{1h}\left(\alpha_{1}+\frac{g_{4h}}{2}\right)\\ \;\;\;\;+e_{1h}^{2}\left(\frac{\alpha_{1}g_{4h}}{2}+\frac{3g_{4h}^{2}}{8}+\frac{\alpha_{1}\left(\alpha_{1}+1\right)}{2}\right)+e_{0h}e_{1h}\left(\alpha_{1}+\frac{g_{4h}}{2}\right)\}\\ \;\;\;\;-k_{2h}\overset{¯}{X_{h}}\left\{e_{2h}-e_{1h}e_{2h}\left(\alpha_{2}+\frac{g_{4h}}{2}\right)\right\}]. \end{array}$$

Using (4.4), the bias of ${\overset{ˆ}{S}}_{Dst}^{2}$ up to first order of approximation is given by

(4.5) $$Bias\left({\overset{ˆ}{S}}_{Dst}^{2}\right)≅-\sum_{h=1}^{L}\theta_{h}W_{h}^{2}\left[S_{yh}^{2}-S_{yh}^{2}k_{1h}D_{h}-k_{2h}G_{h}\right],$$

where

$$D_{h}=\left[1+\theta_{h}\{\lambda_{04h}^{⁎}\left(\frac{3g_{4h}^{2}+4\alpha_{1}\left(g_{4h}+\alpha_{1}+1\right)}{8}\right)-\lambda_{22h}^{⁎}\left(\frac{\alpha_{1}+g_{4h}}{2}\right)\}\right]$$

and

$$G_{h}=\theta_{h}S_{xh}\lambda_{03h}\left(\frac{\alpha_{2}+g_{4h}}{2}\right).$$

The *MSE* of ${\overset{ˆ}{S}}_{Dst}^{2}$ up to the first order of approximation is obtained by squaring both sides of (4.4) and applying expectation, which is given by

(4.6) $$MSE\left({\overset{ˆ}{S}}_{Dst}^{2}\right)≅\sum_{h=1}^{L}\theta_{h}^{2}W_{h}^{4}\left[S_{yh}^{4}+S_{yh}^{4}k_{1h}^{2}A_{h}+k_{2h}^{2}B_{h}-2S_{yh}^{4}k_{1h}D_{h}-2S_{yh}^{2}k_{2h}G_{h}+2S_{yh}^{2}k_{1h}k_{2h}F_{h}\right],$$

where

$$\begin{array}{c} A_{h}=[1+\theta_{h}\{\lambda_{40h}^{⁎}+\lambda_{04h}^{⁎}\left\{{\left(\alpha_{1}+\frac{g_{4h}}{2}\right)}^{2}+\left(\alpha_{1}g_{4h}+\frac{3g_{4h}^{2}}{4}+\frac{\alpha_{1}\left(\alpha_{1}+1\right)}{2}\right)\right\}\\ \;-2\lambda_{22h}^{⁎}\left(2\alpha_{1}+g_{4h}\right)\}], \end{array}$$

$$B_{h}=\theta_{h}S_{xh}^{2}$$

and

$$F_{h}=\theta_{h}S_{xh}\left[\lambda_{03h}(\alpha_{1}+\alpha_{2}+g_{4h})-\lambda_{21h}\right].$$

After minimizing (4.6), the optimal values of $k_{1h}$ and $k_{2h}$ are, respectively provided by

$$k_{1h\left(opt\right)}=\frac{B_{h}D_{h}-F_{h}G_{h}}{A_{h}B_{h}-F_{h}^{2}}$$

and

$$k_{2h\left(opt\right)}=\frac{S_{yh}^{2}\left(A_{h}G_{h}-D_{h}F_{h}\right)}{A_{h}B_{h}-F_{h}^{2}}.$$

The minimum bias and *MSE* for ${\overset{ˆ}{S}}_{Dst}^{2}$ are obtained by putting the optimum values of $k_{1h}$ and $k_{2h}$ into (4.5) and (4.6), which are given by

(4.7) $$Bias{\left({\overset{ˆ}{S}}_{Dst}^{2}\right)}_{min}≅-\sum_{h=1}^{L}\theta_{h}W_{h}^{2}S_{yh}^{2}\left[1-\frac{\left(A_{h}G_{h}^{2}+B_{h}D_{h}^{2}-2D_{h}F_{h}G_{h}\right)}{A_{h}B_{h}-F_{h}^{2}}\right]$$

and

(4.8) $$MSE{\left({\overset{ˆ}{S}}_{Dst}^{2}\right)}_{min}≅\sum_{h=1}^{L}\theta_{h}^{2}W_{h}^{4}S_{yh}^{4}\left[1-\frac{\left(A_{h}G_{h}^{2}+B_{h}D_{h}^{2}-2D_{h}F_{h}G_{h}\right)}{A_{h}B_{h}-F_{h}^{2}}\right].$$

### 4.2 Properties of the suggested estimator-II

Now to discuss the properties of the second suggested estimator (II), we rewrite (4.2) in terms of errors, i.e.

(4.9) $${\overset{ˆ}{S}}_{Dest}^{2}=\sum_{h=1}^{L}\theta_{h}W_{h}^{2}\left[S_{yh}^{2}\left(1+e_{0h}\right)\exp{\left(\frac{-k_{3h}g_{5h}e_{1h}}{2}\left(1+\frac{g_{5h}e_{1h}}{2}\right)\right)}^{-1}\exp{\left(\frac{-k_{4h}g_{6h}e_{2h}}{2}\left(1+\frac{g_{6h}e_{2h}}{2}\right)\right)}^{-1}\right],$$

where $g_{5h}=\frac{\overset{¯}{X_{h}}}{\overset{¯}{X_{h}}+b_{1h}}$ and $g_{6h}=\frac{S_{xh}^{2}}{S_{xh}^{2}+b_{1h}}$.

The following equation is obtained by employing the first-order Taylor series approximation

(4.10) $${\overset{ˆ}{S}}_{Dest}^{2}-\sum_{h=1}^{L}\theta_{h}W_{h}^{2}S_{yh}^{2}≅\sum_{h=1}^{L}\theta_{h}W_{h}^{2}S_{yh}^{2}[e_{0h}-\frac{k_{3h}g_{5h}}{2}e_{1h}-\frac{k_{4h}g_{6h}}{2}e_{2h}+\left(\frac{k_{3h}g_{5h}^{2}}{4}+\frac{k_{3h}^{2}g_{5h}^{2}}{8}\right)e_{1h}^{2}\;\;\;\;\;\;+\left(\frac{k_{4h}g_{6h}^{2}}{4}+\frac{k_{2h}^{2}g_{6h}^{2}}{8}\right)e_{2h}^{2}-\frac{k_{3h}g_{5h}}{2}e_{0h}e_{1h}-\frac{k_{4h}g_{6h}}{2}e_{0h}e_{2h}+\frac{k_{3h}k_{4h}g_{5h}g_{6h}}{2}e_{1h}e_{2h}].$$

Using (4.10), the bias of ${\overset{ˆ}{S}}_{Dest}^{2}$ is given by

(4.11) $$Bias\left({\overset{ˆ}{S}}_{Dest}^{2}\right)≅\sum_{h=1}^{L}\theta_{h}^{2}W_{h}^{2}S_{yh}^{2}[\left(\frac{k_{3h}g_{5h}^{2}}{4}+\frac{k_{3h}^{2}g_{5h}^{2}}{8}\right)\lambda_{04}^{⁎}+\left(\frac{k_{4h}g_{6h}^{2}}{4}+\frac{k_{4h}^{2}g_{6h}^{2}}{8}\right)C_{xh}^{2}-\frac{k_{3h}g_{5h}}{2}\lambda_{22}^{⁎}\;\;\;\;-\frac{k_{4h}g_{6h}}{2}C_{xh}\lambda_{21h}+\frac{k_{3h}k_{4h}g_{5h}g_{6h}}{2}C_{xh}\lambda_{03h}].$$

The *MSE* of ${\overset{ˆ}{S}}_{Dest}^{2}$ up to the first order of approximation is obtained by squaring both sides of (4.10) and applying expectation, which is given by

(4.12) $$MSE\left({\overset{ˆ}{S}}_{Dest}^{2}\right)≅\sum_{h=1}^{L}\theta_{h}^{3}W_{h}^{4}S_{yh}^{4}\left[\lambda_{40h}^{⁎}+\frac{k_{3h}^{2}g_{5h}^{2}}{4}\lambda_{04h}^{⁎}+\frac{k_{4h}^{2}g_{6h}^{2}}{4}C_{xh}^{2}-k_{3h}g_{5h}\lambda_{22h}^{⁎}-k_{4h}g_{6h}C_{xh}\lambda_{21h}+\frac{k_{3h}k_{4h}g_{5h}g_{6h}}{2}C_{xh}\lambda_{03h}\right].$$

We can rewrite the bias and *MSE* for ${\overset{ˆ}{S}}_{Dest}^{2}$ by putting the known values of $\left(k_{3h},k_{4h}\right)$ into (4.11) and (4.12), which are given by

(4.13) $$Bias\left({\overset{ˆ}{S}}_{Dest}^{2}\right)≅\sum_{h=1}^{L}\theta_{h}^{2}W_{h}^{2}S_{yh}^{2}\left[\frac{3}{8}\left(g_{5h}^{2}\lambda_{04}^{⁎}+g_{6h}^{2}C_{xh}^{2}\right)-\frac{1}{2}\left(g_{5h}\lambda_{22}^{⁎}+g_{6h}C_{xh}\lambda_{21h}-g_{5h}g_{6h}C_{xh}\lambda_{03h}\right)\right]$$

and

(4.14) $$MSE\left({\overset{ˆ}{S}}_{Dest}^{2}\right)≅\sum_{h=1}^{L}\theta_{h}^{3}W_{h}^{4}S_{yh}^{4}\left[\lambda_{40h}^{⁎}+\frac{1}{4}\left(g_{5h}^{2}\lambda_{04h}^{⁎}+g_{6h}^{2}C_{xh}^{2}-4g_{5h}\lambda_{22h}^{⁎}-4g_{6h}C_{xh}\lambda_{21h}+2g_{5h}g_{6h}C_{xh}\lambda_{03h}\right)\right].$$

## 5 Mathematical comparison

This section covers the comparisons of the proposed estimators ${\overset{ˆ}{S}}_{Dst}^{2}$ and ${\overset{ˆ}{S}}_{Dest}^{2}$, with other existing estimators ${\overset{ˆ}{S}}_{yst}^{2},{\overset{ˆ}{S}}_{Rst}^{2},{\overset{ˆ}{S}}_{lrst}^{2},{\overset{ˆ}{S}}_{BTst}^{2},{\overset{ˆ}{S}}_{USst}^{2}$, and ${\overset{ˆ}{S}}_{KCist}^{2}$.

### 5.1 Suggested estimator-I

Condition (i): By (3.1) and (4.8)

$$Var({\overset{ˆ}{S}}_{yst}^{2})>MSE{\left({\overset{ˆ}{S}}_{Dst}^{2}\right)}_{min}\text{ if}$$

$$\sum_{h=1}^{L}\theta_{h}^{2}W_{h}^{4}S_{yh}^{4}\left[\theta_{h}\lambda_{40h}^{⁎}-1+\left(\frac{A_{h}G_{h}^{2}+B_{h}D_{h}^{2}-2_{h}D_{h}F_{h}G_{h}}{A_{h}B_{h}-F_{h}^{2}}\right)\right]>0.$$

Condition (ii): By (3.4) and (4.8)

$$MSE({\overset{ˆ}{S}}_{Rst}^{2})>MSE{\left({\overset{ˆ}{S}}_{Dst}^{2}\right)}_{min}\text{ if}\sum_{h=1}^{L}\theta_{h}^{2}W_{h}^{4}S_{yh}^{4}\left[\theta_{h}\left(\lambda_{40h}^{⁎}+\lambda_{04h}^{⁎}-2\lambda_{22h}^{⁎}\right)-1+\left(\frac{A_{h}G_{h}^{2}+B_{h}D_{h}^{2}-2_{h}D_{h}F_{h}G_{h}}{A_{h}B_{h}-F_{h}^{2}}\right)\right]>0.$$

Condition (iii): By (3.6) and (4.8)

$$MSE({\overset{ˆ}{S}}_{lrst}^{2})>MSE{\left({\overset{ˆ}{S}}_{Dst}^{2}\right)}_{min}\text{ if}\sum_{h=1}^{L}\theta_{h}^{2}W_{h}^{4}S_{yh}^{4}\left[\theta_{h}\lambda_{40h}^{⁎}\left(1-\rho_{h}^{⁎2}\right)-1+\left(\frac{A_{h}G_{h}^{2}+B_{h}D_{h}^{2}-2_{h}D_{h}F_{h}G_{h}}{A_{h}B_{h}-F_{h}^{2}}\right)\right]>0.$$

Condition (iv): By (3.9) and (4.8)

$$MSE({\overset{ˆ}{S}}_{BTst}^{2})>MSE{\left({\overset{ˆ}{S}}_{Dst}^{2}\right)}_{min}\text{ if}\sum_{h=1}^{L}\theta_{h}^{2}W_{h}^{4}S_{yh}^{4}\left[\theta_{h}\left(\lambda_{40h}^{⁎}+\frac{\lambda_{04h}^{⁎}}{4}-\lambda_{22h}^{⁎}\right)-1+\left(\frac{A_{h}G_{h}^{2}+B_{h}D_{h}^{2}-2_{h}D_{h}F_{h}G_{h}}{A_{h}B_{h}-F_{h}^{2}}\right)\right]>0.$$

Condition (v): By (3.12) and (4.8)

$$MSE({\overset{ˆ}{S}}_{USst}^{2})>MSE{\left({\overset{ˆ}{S}}_{Dst}^{2}\right)}_{min}\text{ if}\sum_{h=1}^{L}\theta_{h}^{2}W_{h}^{4}S_{yh}^{4}\left[\theta_{h}\left(\lambda_{40h}^{⁎}+g_{0h}^{2}\lambda_{04h}^{⁎}-2g_{0h}\lambda_{22h}^{⁎}\right)-1+\left(\frac{A_{h}G_{h}^{2}+B_{h}D_{h}^{2}-2_{h}D_{h}F_{h}G_{h}}{A_{h}B_{h}-F_{h}^{2}}\right)\right]>0.$$

Condition (vi): By (3.17) and (4.8)

$$MSE({\overset{ˆ}{S}}_{KCist}^{2})>MSE{\left({\overset{ˆ}{S}}_{Dst}^{2}\right)}_{min}\text{ if}\sum_{h=1}^{L}\theta_{h}^{2}W_{h}^{4}S_{yh}^{4}\left[\theta_{h}\left(\lambda_{40h}^{⁎}+g_{ih}^{2}\lambda_{04h}^{⁎}-2g_{ih}\lambda_{22h}^{⁎}\right)-1+\left(\frac{A_{h}G_{h}^{2}+B_{h}D_{h}^{2}-2_{h}D_{h}F_{h}G_{h}}{A_{h}B_{h}-F_{h}^{2}}\right)\right]>0.$$

### 5.2 Suggested estimator-II

Condition (vii): By (3.1) and (4.14)

$$Var({\overset{ˆ}{S}}_{yst}^{2})>MSE\left({\overset{ˆ}{S}}_{Dest}^{2}\right)\text{ if}$$

$$\sum_{h=1}^{L}\theta_{h}^{3}W_{h}^{4}S_{yh}^{4}\left[g_{5h}\lambda_{22h}^{⁎}+g_{6h}C_{xh}\lambda_{21h}\right]>\frac{1}{4}\sum_{h=1}^{L}\theta_{h}^{3}W_{h}^{4}S_{yh}^{4}\left[g_{5h}^{2}\lambda_{04h}^{⁎}+g_{6h}^{2}C_{xh}^{2}+2g_{5h}g_{6h}C_{xh}\lambda_{03h}\right].$$

Condition (viii): By (3.4)and (4.14)

$$MSE({\overset{ˆ}{S}}_{Rst}^{2})>MSE\left({\overset{ˆ}{S}}_{Dest}^{2}\right)\text{ if}\sum_{h=1}^{L}\theta_{h}^{3}W_{h}^{4}S_{yh}^{4}\left[\lambda_{04h}^{⁎}+\lambda_{22h}^{⁎}\left(g_{5h}-2\right)+g_{6h}C_{xh}\lambda_{21h}\right]>\frac{1}{4}\sum_{h=1}^{L}\theta_{h}^{3}W_{h}^{4}S_{yh}^{4}\left[g_{5h}^{2}\lambda_{04h}^{⁎}+g_{6h}^{2}C_{xh}^{2}+2g_{5h}g_{6h}C_{xh}\lambda_{03h}\right].$$

Condition (ix): By (3.6) and (4.14)

$$MSE({\overset{ˆ}{S}}_{lrst}^{2})>MSE\left({\overset{ˆ}{S}}_{Dest}^{2}\right)\text{ if}\sum_{h=1}^{L}\theta_{h}^{3}W_{h}^{4}S_{yh}^{4}\left[g_{5h}\lambda_{22h}^{⁎}+g_{6h}C_{xh}\lambda_{21h}-\lambda_{04h}^{⁎}\rho_{h}^{⁎2}\right]>\frac{1}{4}\sum_{h=1}^{L}\theta_{h}^{3}W_{h}^{4}S_{yh}^{4}\left[g_{5h}^{2}\lambda_{04h}^{⁎}+g_{6h}^{2}C_{xh}^{2}+2g_{5h}g_{6h}C_{xh}\lambda_{03h}\right].$$

Condition (x): By (3.9) and (4.14)

$$MSE({\overset{ˆ}{S}}_{BTst}^{2})>MSE\left({\overset{ˆ}{S}}_{Dest}^{2}\right)\text{ if}\sum_{h=1}^{L}\theta_{h}^{3}W_{h}^{4}S_{yh}^{4}\left[\lambda_{04h}^{⁎}+4\lambda_{22h}^{⁎}\left(g_{5h}-1\right)+4g_{6h}C_{xh}\lambda_{21h}\right]>\sum_{h=1}^{L}\theta_{h}^{3}W_{h}^{4}S_{yh}^{4}\left[g_{5h}^{2}\lambda_{04h}^{⁎}+g_{6h}^{2}C_{xh}^{2}+2g_{5h}g_{6h}C_{xh}\lambda_{03h}\right].$$

Condition (xi): By (3.12) and (4.14)

$$MSE({\overset{ˆ}{S}}_{USst}^{2})>MSE\left({\overset{ˆ}{S}}_{Dest}^{2}\right)\text{ if}\sum_{h=1}^{L}\theta_{h}^{3}W_{h}^{4}S_{yh}^{4}\left[g_{0h}^{2}\lambda_{04h}^{⁎}+\lambda_{22h}^{⁎}\left(g_{5h}-2g_{0h}\right)+g_{6h}C_{xh}\lambda_{21h}\right]>\frac{1}{4}\sum_{h=1}^{L}\theta_{h}^{3}W_{h}^{4}S_{yh}^{4}\left[g_{5h}^{2}\lambda_{04h}^{⁎}+g_{6h}^{2}C_{xh}^{2}+2g_{5h}g_{6h}C_{xh}\lambda_{03h}\right].$$

Condition (xiii): By (3.17) and (4.14)

$$MSE({\overset{ˆ}{S}}_{KCist}^{2})>MSE\left({\overset{ˆ}{S}}_{Dest}^{2}\right)\text{ if}\sum_{h=1}^{L}\theta_{h}^{3}W_{h}^{4}S_{yh}^{4}\left[g_{ih}^{2}\lambda_{04h}^{⁎}+\lambda_{22h}^{⁎}\left(g_{5h}-2g_{ih}\right)+g_{6h}C_{xh}\lambda_{21h}\right]>\frac{1}{4}\sum_{h=1}^{L}\theta_{h}^{3}W_{h}^{4}S_{yh}^{4}\left[g_{5h}^{2}\lambda_{04h}^{⁎}+g_{6h}^{2}C_{xh}^{2}+2g_{5h}g_{6h}C_{xh}\lambda_{03h}\right].$$

## 6 Numerical comparison

In this section, we compare the percent relative efficiency (PRE) of different estimators using simulated and real data sets in order to assess the effectiveness of the suggested estimators.

### 6.1 Simulation study

In order to verify the theoretical results discussed in Section 5, we utilize the idea from [12], [13] to conduct a simulation study to determine the effectiveness of the suggested estimators based on known extreme values of the auxiliary variable. The following probability distributions can be used to artificially obtain six different populations for auxiliary variable X:

• Population 1: $X∼Gamma(\eta_{1}=4,\eta_{2}=6)$,
• Population 2: $X∼Gamma(\eta_{1}=8,\eta_{2}=10)$,
• Population 3: X∼Exponential(λ=3),
• Population 4: X∼Exponential(λ=7),
• Population 5: $X∼Uniform(\nu_{1}=0,\nu_{2}=1)$,
• Population 6: $X∼Uniform(\nu_{1}=3,\nu_{2}=5)$.

The variable of interest *Y* is then computed as

$$Y=r_{yx}\times X+e,$$

where e∼N(0,1) denotes the error term, and $r_{yx}=0.80$ is the correlation coefficient between (y,x).

To find the (*PREs*) of the proposed estimators, we adopted the following procedures in R- software:

**Step 1:** To generate a population of size 1000, we firstly employ specific types of probability distributions. This population is then divided into two different strata to compute various values of recommended and existing estimators through the use of stratified random sampling methods.

**Step 2:** We obtain the population total from Step 1, as well as the smallest and largest values of the auxiliary variable. Additionally, we calculate the optimal values of the suggested estimators' unknown constants.

**Step 3:** To obtain different sample sizes for each population, we apply simple random sampling without replacement *(*SRSWOR). The various sample sizes are defined as (n = 20%; 30%; 40% of N).

**Step 4:** For each sample size, find the PREs values of all the estimators discussed in this article. **Step 5:** Following 50000 repetitions of steps 3 and 4, the results for artificial populations are displayed in Table 2, while the results for real data sets are given in Table 3.

**Table 2:** Percent relative efficiency (PRE) of the estimators using the artificial populations.

Estimator	*Data-I*	*Data-II*	*Data-III*	*Data-IV*	*Data-V*	*Data-VI*
[1] ${\overset{ˆ}{S}}_{y}^{2}$	100	100	100	100	100	100
[2] ${\overset{ˆ}{S}}_{R}^{2}$	126.587	109.523	113.145	108.526	120.560	107.745
[3] ${\overset{ˆ}{S}}_{lr}^{2}$	135.635	123.145	125.315	122.302	122.705	108.565
[4] ${\overset{ˆ}{S}}_{BT}^{2}$	142.407	127.548	128.100	124.240	121.445	106.404
[5] ${\overset{ˆ}{S}}_{US}^{2}$	143.289	126.905	132.409	126.526	119.078	109.425
[6] ${\overset{ˆ}{S}}_{KC1}^{2}$	145.526	135.858	136.620	128.789	124.589	112.408
[7] ${\overset{ˆ}{S}}_{KC2}^{2}$	145.552	135.058	135.556	128.002	123.164	110.528
[8] ${\overset{ˆ}{S}}_{KC3}^{2}$	144.203	134.889	133.427	129.328	121.664	116.548
[9] ${\overset{ˆ}{S}}_{D1s}^{2}$	573.179	673.891	435.950	512.369	695.289	1201.333
[10] ${\overset{ˆ}{S}}_{D2s}^{2}$	1589.369	1062.910	1483.920	1610.089	1991.280	2035.280
[11] ${\overset{ˆ}{S}}_{D3s}^{2}$	360.978	660.737	723.625	885.069	761.132	1211.036
[12] ${\overset{ˆ}{S}}_{D4s}^{2}$	505.576	605.614	523.353	689.701	710.369	910.268
[13] ${\overset{ˆ}{S}}_{D5s}^{2}$	419.616	522.666	370.323	400.104	625.712	1095.498
[14] ${\overset{ˆ}{S}}_{D6s}^{2}$	430.448	330.184	310.688	427.102	439.259	668.948
[15] ${\overset{ˆ}{S}}_{D7s}^{2}$	883.149	983.921	643.679	683.125	989.949	1259.309
[16] ${\overset{ˆ}{S}}_{D8s}^{2}$	5284.562	4282.380	3348.910	3589.014	4410.720	4709.460
[17] ${\overset{ˆ}{S}}_{Des}^{2}$	224.668	210.798	204.139	215.891	207.724	200.589

**Table 3:** Percent relative efficiency of different estimators with respect to ${\overset{ˆ}{S}}_{yst}^{2}$.

Estimator	Data 1	Data 2	Data 3
[1] ${\overset{ˆ}{S}}_{yst}^{2}$	100	100	100
[2] ${\overset{ˆ}{S}}_{Rst}^{2}$	124.425	203.430	152.542
[3] ${\overset{ˆ}{S}}_{lrst}^{2}$	260.593	374.638	81.347
[4] ${\overset{ˆ}{S}}_{BTst}^{2}$	165.547	211.249	174.056
[5] ${\overset{ˆ}{S}}_{USst}^{2}$	112.912	115.363	160.266
[6] ${\overset{ˆ}{S}}_{KC1st}^{2}$	124.425	203.432	152.823
[7] ${\overset{ˆ}{S}}_{KC2st}^{2}$	124.425	203.430	152.610
[8] ${\overset{ˆ}{S}}_{KC3st}^{2}$	135.935	206.336	192.035
[9] ${\overset{ˆ}{S}}_{D1st}^{2}$	407.697	631.073	250.702
[10] ${\overset{ˆ}{S}}_{D2st}^{2}$	412.355	507.307	259.714
[11] ${\overset{ˆ}{S}}_{D3st}^{2}$	398.825	592.214	270.539
[12] ${\overset{ˆ}{S}}_{D4st}^{2}$	308.385	554.749	282.115
[13] ${\overset{ˆ}{S}}_{D5st}^{2}$	312.365	528.876	291.913
[14] ${\overset{ˆ}{S}}_{D6st}^{2}$	330.870	556.546	316.095
[15] ${\overset{ˆ}{S}}_{D7st}^{2}$	353.406	596.862	354.497
[16] ${\overset{ˆ}{S}}_{D8st}^{2}$	605.653	1030.068	580.205
[17] ${\overset{ˆ}{S}}_{Dest}^{2}$	273.209	413.294	244.505

Finally, obtain the *MSEs* and *PREs* for each estimator over all replications using the following formulas:

$$MSE{\left({\overset{ˆ}{S}}_{k}^{2}\right)}_{\min}=\frac{\sum_{g=1}^{50000}{\left({\overset{ˆ}{S}}_{k}^{2}-S_{yst}^{2}\right)}^{2}}{50000}$$

and

$$PRE=\frac{V({\overset{ˆ}{S}}_{yst}^{2})}{MSE{\left({\overset{ˆ}{S}}_{k}^{2}\right)}_{min}}\times 100,$$

where k is one of Rst,lrst,BTst,USst,KC1st,KC2st,KC3st,Dist(i=1,2,…,8,e).

### 6.2 Numerical examples

We compared the percent relative efficiency (PREs) of different estimators using real datasets in order to assess the performance of the suggested classes of estimators. The data-sets are listed below.

**Data 1.** [**Source:** [7], p. 135]

*Y*: The total number of learners enrolled in 2012,

*X*: The total number of elementary and secondary schools in 2012

**Group 1**: Comprises the divisions of Sargodha, Gujranwala, Rawalpindi, and Lahore.

**Group 2**: Comprises the divisions of Multan, Bahawalpur, Faisalabad, D.G Khan, and Sahiwal.

The summary statistics are as follows:

$$\begin{array}{c} N_{1}=18,N_{1}=18,n_{1}=8,n_{2}=8,\overset{¯}{X_{1}}=962.056,\overset{¯}{X_{2}}=1146,\overset{¯}{Y_{1}}=162979.3,\overset{¯}{Y_{2}}=134458,\\ X_{M_{1}}=1530,X_{M_{2}}=2370,X_{m1}=388,X_{m2}=582,S_{x1}=307.953,S_{x2}=469.931,S_{y1}=255887.7,\\ S_{y2}=50235.82,C_{x1}=0.320,C_{x2}=0.409,C_{y1}=1.571,C_{y2}=0.374,\rho_{yx1}=0.145,\rho_{yx2}=0.787,\\ \lambda_{401}=2625,\lambda_{402}=2240,\lambda_{041}=3237,\lambda_{042}=2558,\lambda_{031}=1548,\lambda_{032}=2049,\lambda_{211}=1298,\\ \lambda_{212}=1200,\lambda_{221}=1568,\lambda_{222}=1807. \end{array}$$

**Data 2.** [**Source:** [7], p. 226]

*Y*: Employment levels by departments in 2012,

*X*: The total number of factories listed by departments in 2012.

Two groups are generated from the data:

**Group 1**: Comprises the divisions of Sargodha, Gujranwala, Rawalpindi, and Lahore.

**Group 2**: Comprises the divisions of Multan, Bahawalpur, Faisalabad, D.G Khan, and Sahiwal.

The summary statistics are as follows:

$$\begin{array}{c} N_{1}=18,N_{1}=18,n_{1}=8,n_{2}=8,\overset{¯}{X_{1}}=414.556,\overset{¯}{X_{2}}=257,\overset{¯}{Y_{1}}=85572.11,\overset{¯}{Y_{2}}=19293.61,\\ X_{M_{1}}=2055,X_{M_{2}}=1674,X_{m1}=24,X_{m2}=52,S_{x1}=521.675,S_{x2}=365.696,S_{y1}=248216,\\ S_{y2}=37979.33,C_{x1}=1.258,C_{x2}=1.423,C_{y1}=2.901,C_{y2}=1.969,\rho_{yx1}=0.337,\rho_{yx2}=0.976,\\ \lambda_{401}=3270,\lambda_{402}=2542,\lambda_{041}=3345,\lambda_{042}=2388,\lambda_{031}=944,\lambda_{032}=739,\lambda_{211}=1267,\\ \lambda_{212}=988,\lambda_{221}=2398,\lambda_{222}=2246. \end{array}$$

**Data 3.** (**Source:** [10], p. 24)

*Y*: Food costs associated with the work,

*X*: Weekly income of the household.

Two groups are generated from the data, and the summary statistics are as follows:

$$\begin{array}{c} N_{1}=18,N_{1}=18,n_{1}=8,n_{2}=8,\overset{¯}{Y_{1}}=27.49,\overset{¯}{Y_{2}}=20.82,\overset{¯}{X_{1}}=72.55,\overset{¯}{X_{2}}=60.87,\\ X_{M_{1}}=95,X_{M_{2}}=75,X_{m1}=28,X_{m2}=15,S_{y1}=10.13,S_{y2}=12.75,S_{x1}=10.58,\\ S_{x2}=8.98,C_{x1}=0.155,C_{x2}=0.142,C_{y1}=0.376,C_{y2}=0.269,\rho_{yx1}=0.337,\\ \rho_{yx2}=0.496,\lambda_{401}=2.55,\lambda_{402}=4.142,\lambda_{041}=2.845,\lambda_{042}=3.934,\lambda_{031}=4.544,\\ \lambda_{032}=1.239,\lambda_{211}=4.542,\lambda_{212}=2.488,\lambda_{221}=3.158,\lambda_{222}=1.384. \end{array}$$

For efficiency comparisons, we use the following formula:

$$PRE=\frac{V({\overset{ˆ}{S}}_{yst}^{2})}{MSE({\overset{ˆ}{S}}_{k}^{2})}\times 100$$

where k is one of Rst,lrst,BTst,USst,KC1st,KC2st,KC3st,Dist(i=1,2,…,8,e). Table 3 summarizes the percent relative efficiency for three different data sets.

## 7 Conclusion

We proposed some classes of efficient estimators for calculating the finite population variance in this article. These estimators make use of the auxiliary variable's known extreme values. In Section 5, we discussed theoretical conditions that show the better efficiency of the proposed estimators, enabling us to compare their characteristics with those of existing estimators. We performed a simulation study and examined a number of empirical datasets in order to validate these conditions. The proposed estimators are consistently better than existing estimators in terms of PREs, according to the results displayed in Table 2. The empirical results in Table 3 further support this observation and validate the theoretical conclusions in Section 5.

The suggested estimators ${\overset{ˆ}{S}}_{Dist}^{2}$ (i=1,2,3,…,8,e) show higher efficiency compared to the other existing estimators, and ${\overset{ˆ}{S}}_{D8st}^{2}$ is especially selected among these proposed estimators because of its minimum *MSE*, according to both the simulation and empirical results.

However, we examined the characteristics of the proposed effective classes of estimators using a stratified random sampling approach. Our results can be useful in identifying the more efficient estimators that can provide the lowest MSEs. It is also possible to offer some novel estimators in the two-phase stratified sampling approach. It is also an interesting topic for further research.

## CRediT authorship contribution statement

**Umer Daraz:** Writing – review & editing, Writing – original draft, Validation, Software, Resources, Project administration, Methodology, Formal analysis, Data curation, Conceptualization. **Jinbiao Wu:** Visualization, Validation, Supervision, Resources, Investigation, Formal analysis, Data curation, Conceptualization. **Mohammed Ahmed Alomair:** Visualization, Resources, Project administration, Investigation, Funding acquisition, Formal analysis. **Luai Abdulla Aldoghan:** Visualization, Resources, Investigation, Funding acquisition.

## Declaration of Competing Interest

The authors declare that they have no known competing financial interests or personal relationships that could have appeared to influence the work reported in this paper.
